# Genome Sequence of *Klebsiella quasipneumoniae* subsp. *similipneumoniae* MB373, an Effective Bioremediator

**DOI:** 10.1128/genomeA.01068-16

**Published:** 2016-09-29

**Authors:** Fozia Aslam, Azra Yasmin, Torsten Thomas

**Affiliations:** a Department of Environmental Sciences, Microbiology and Biotechnology Research Lab, Fatima Jinnah Women University, Rawalpindi, Pakistan; b Centre for Marine Bio-innovation & School of Biological, Earth and Environmental Sciences, The University of New South Wales, Sydney, New South Wales, Australia

## Abstract

*Klebsiella quasipneumoniae* subsp. *similipneumoniae* MB373 was isolated from effluent of the Hattar Industrial Estate, Haripur, Pakistan. *K. quasipneumoniae* subsp. *similipneumoniae* has few cultivated/characterized members so far. Whole-genome sequencing revealed its potential for metal and toxin resistance, which further elucidated various enzymatic processes for the degradation of xenobiotics, illuminating its bioremediation applications.

## GENOME ANNOUNCEMENT

Abundant contaminants produced by industrial activities have resulted in the need to remediate the affected environment. For example, industrial effluents often contain various organic and inorganic pollutants, including heavy metals that have been shown to represent a risk to human health (1). Microorganisms isolated from such contaminated environments have a potential role in the remediation processes, as they often contain a diversity of genes that are involved in the degradation of xenobiotics and resistance to heavy metals or toxins (2). In this context, several bacterial genera have been reported for efflux-mediated metal tolerance/detoxification mechanisms, which have been acquired through horizontal gene transfer (3, 4).

Here, we present the genome sequence of *Klebsiella quasipneumoniae* subsp. *similipneumoniae* MB373. The strain was chosen for its high resistance against various heavy metals and antibiotics as well as for its ability to utilize and degrade organic compounds. Genomic DNA extraction and whole-genome sequencing were performed with Illumina MiSeq technology. The sequence reads obtained were of good quality, as determined by FastQC (5). Afterward, SPAdes version 3.1.0 yielded an assembly of 39 contigs (6). The *N*_50_ contig size was approximately 368,603 bp, showing genome coverage of about 104.0×. The assembled genome consists of 5,440,152 bp, with 57.5% G+C content.

Gene prediction and annotation were done with Rapid Annotations using Subsystems Technology (RAST) and Prokaryotic Genome Annotation Pipeline (PGAP) by NCBI (http://www.ncbi.nlm.nih.gov/genome/annotation_prok). According to PGAP, the draft genome comprised 5,343 coding sequences, with 5,136 protein-coding genes and 105 pseudogenes. There had been 102 RNA genes, out of which 13 were identified for rRNAs and 76 for tRNAs using RNAmmer (7) and tRNAscan SE (8), respectively.

RAST server results revealed 5,203 genes that were related to 583 SEED subsystems, with 58 possibly missing genes (9). Most genes were designated to the metabolism of carbohydrates (807), amino acids, and/or their derivatives (572). One hundred eighty-one genes were allocated for stress response, including universal stress protein, phage shock protein (psp) operon, and an Hfl operon. One hundred nineteen genes were assigned to the functions associated with antibiotics and toxic chemical resistances. Among metal tolerance, 22 genes were annotated for proteins related to Co-Zn-Cd, whereas nine, two, and 11 genes were identified for copper, chromium, and arsenic tolerance mechanisms, respectively.

The genome possessed 130 genes for cell regulation and signaling, which encoded BOX elements in streptococci, quorum sensing (auto inducer 2 [AI-2] transport and processing *lsrACDBFGE* operon), biofilm adhesion biosynthesis, and detoxification of tellurium compounds, etc. Furthermore, functional analysis revealed 80 genes for enzymes effectively participating in the metabolism of various aromatic compounds comprising peripheral catabolic pathways of salicylate ester, quinate, benzoate, *p*-hydroxybenzoate, chloroaromatic, as well as anaerobic decarboxylation of hydroxyaromatic compounds. Additionally, 50 genes were designated for the metabolism of central aromatic intermediates of salicylate and gentisate catabolism (six genes), catechol (eight genes), and the protocatechuate (17 genes) branch of the beta-ketoadipate pathway.

In short, the genome sequence of strain MB373 revealed various genes coding for enzymes that could be involved in the biotransformation of organic pollutants as well as resistance and detoxification of heavy metals. This makes the strain a potential candidate for bioremediation processes for environments polluted with industrial effluents.

### Accession number(s).

The whole-genome shotgun project of *Klebsiella quasipneumoniae* subsp. *similipneumoniae* MB373 has been deposited at DDBJ/ENA/GenBank under accession no. LYSU00000000. The version of this paper is version LYSU01000000.
